# Propolis Contra Pharmacological Interventions in Bees

**DOI:** 10.3390/molecules27154914

**Published:** 2022-08-01

**Authors:** Joanna Wojtacka

**Affiliations:** Department of Veterinary Public Health, Faculty of Veterinary Medicine, University of Warmia and Mazury in Olsztyn, 10-718 Olsztyn, Poland; joanna.wojtacka@uwm.edu.pl; Tel.: +48-89-523-33-31

**Keywords:** propolis, honey bee, veterinary medicinal products, VMP, MRL, residues, varroacides, pesticides, acaricides, veterinary public health

## Abstract

In addition to wax, propolis is a mixture of resins, terpenes, and etheric and aromatic oils. This composition supports its very strong biochemical activity that affects bee health. Bee colonies are externally exposed to the activity of other different pharmacologically active substances and toxic agents used in beekeeping procedures, veterinary interventions, and the environment. Even if free form common diseases, they may suffer from parasites or toxins. In any such case the abundance and variety of honeyflow, besides proper therapy, is crucial for the maintenance of bee health. Propolis itself cannot be considered as food but can be considered as micro-nutrients for bees. This is due to the fact that some of its compounds may penetrate different bee products, and this way be consumed by bees and their larvae, while stored in the hive. This perspective shows propolis as natural agent reducing the toxicity of pyrethroid acaricides, stimulating production of detoxification enzymes, enhancing the action of antibiotics, and increasing expression of genes that encode proteins responsible for detoxication. The aim of this review is to summarize current data on the possible impact on veterinary public health of the introduction into propolis of residues of pharmacological agents approved in the EU for use in the treatment of bee colonies and their environment.

## 1. Introduction

Resin collection and propolis production are incorporated in honey bees’ instinctive behavior. Some roles of propolis in bees colonies have been well studied, i.e., hive construction and preservation, sealing material, or material for encapsulation of invaders’ cadavers [1]. Propolis enables the comb to carry the load of nectar and honeydew. Its share in the comb composition is estimated to be approximately 5–10%. It reinforces the whole structure of the comb and is present in brood cells and honey cells in the form of a thin layer [2]. Results have confirmed the evolutionarily important function of propolis in a form of social immunity, showing that resins within the nest decrease investment in the immune function of adult bees [3]. At least six main propolis types have been classified based on phenolic-resinous fraction studies, i.e., poplar, birch, green, red, “pacific”, and “canarian” propolis [4]. However, the exact biochemical role and significance of propolis still require analysis. It has been confirmed that the quantity and quality of propolis vary depending on the region [5] and the variety of the collecting bee, even if the harvest applies to one region [6,7]. Lack of natural standardization makes propolis an interesting subject for research in terms of its impact on human and bee health and the quality of bee products. At the same time, the lack of legal standardization makes propolis a difficult subject for validation in terms of assurance of veterinary public health. As a product imported to the EU it must conform to generally binding requirements for bee products, and no legal act at the level of the EU regulator applies particularly to propolis as it does to honey. In some EU countries, there are so-called technical norms regulating the general and organoleptic quality of propolis. Not much is known about their content as many of them are in national languages and their observance is voluntary, as is the case with the Polish standard [8]. Companies purchasing propolis from the market for further processing follow their own company standards, in order to fulfill good manufacturing and good hygiene practices. This approach is crucial because propolis is a subject of worldwide turnover. An average 1800 to 2400 tons of propolis are produced annually. Indonesia is the main exporter of propolis, with an estimated 61.4% share globally. In Europe, Spain is the main exporter of propolis, ranking fifth in the world with a share of 1.4% [9]. Basic evaluation of propolis applies to recognition of defects, i.e., the presence of wax flukes or other worms at all stages of development, asphalt particles, window putty, presence of mold, or an unusual smell. Propolis meeting the organoleptic requirements should have a yellow to dark brown color, often with a greenish or reddish tinge, a pleasant, balsamic smell, and its consistency, depending on the temperature, can be hard and brittle (below 20 °C) or plastic (above 20 °C). In Poland, propolis is divided into two quality classes, which are defined on the basis of the presence of substances and compounds insoluble in 95% ethanol, verified after filtering ethanolic propolis extract through a filter paper. These substances include: wax, splinters, dead bees, and other solid contaminants. The higher class of propolis can contain up to 30%, and the lower class up to 50% of these substances. Removing wax from propolis assures its purity, but also directly translates into the content of undesirable fat-soluble substances including drug residues.

During international trade when entering the territory of EU, edible propolis must be accompanied by a health certificate assuring its conformation to the official model for honey and other apiculture products intended for human consumption [10]. Information included in the document applies to traceability, conditions of transport, packaging, and public health attestation. The latter is closely related to the conditions that guarantee compliance with maximum residue limits (MRLs) for pesticides laid down in EU law [11,12]. Nevertheless, a new legal basis should be elaborated in order to establish adequate and stricter controls on the quality of edible propolis. Such formal regulation issued at EU level would finally define propolis, which, like other apiculture products except for honey, lacks formal definition. There is no clear legal indication whether propolis is a product of plant or animal origin, although the latter is obvious when the way of collection, transporting to the hive, and utilization by bees is taken into account. An accepted definition would be a milestone for EU and national regulators for elaborating specific requirements in relation to the quality and safety of propolis, and amending existing legal acts in order to protect human health.

Bee health and the safety of bee products directly depend on environmental impact and beekeeping procedures, especially including pharmacological interventions when the use of drugs is needed [13]. Veterinary medicines, also known as medicinal products for veterinary use, veterinary drugs, or veterinary medicinal products (VMPs), are substances or combinations of substances to treat, prevent or diagnose disease in animals including honey bees [14]. Their residues in foodstuffs are monitored and MRLs have been established. As the use of VMPs in the bee sector must comply with the EU law, treatment of bee colonies with antibiotics is banned. Thus, no MRLs for antibiotic residues in bee products have been established [15], and the only drugs distributed under veterinary supervision are those for varroosis and nosemosis control, according to the authorization of national regulators. Based on new EU regulation [14], three databases were created in order to improve access to and share information on VMPs. Union Product Database [16] holds information on all VMPs authorized in the EU by the European Commission (EC) and the competent authorities of the Member States. VMPs authorized for use in bee colonies comprise synthetic and natural acaricides, and verilopam (3-(paminophenethyl)-2,3,4,5-tetrahydro-7,8-dimethoxy-1H-3-benzazepine), a potent analgesic in the form of coarse spray (Table 1).

VMPs for treatment of bee colonies are available in different pharmacological forms. These include in-hive preparations, i.e., bee-hive strips, bee-smoke strips, products for topical treatments in the form of spray, fumigation, nebulization and inhalation, and formulas for oral application in sugar syrup or sugar cake. Such a wide variety of particular substance distribution reflects its pharmacokinetics, pharmacodynamics, and pharmacogenetics. Honey bees carry out the detoxification process in three phases. However, despite potential natural exposure to a broad diversity of phytochemicals, their genome is characterized by a paucity of genes associated with detoxification [17]. As they lack liver, kidneys, spleen, and lungs the role of excretion is taken over by the fat body, the Malphigian tubules, the hindgut, and the cuticle [18]. The negative effects of acaricides on honey bees may be discussed, with direct post-application effects corelated with such factors as the age of the bees, stress levels, temperature, physiological differences (queen/worker), mobility, and acaricide dose. Obviously, the method of application has significant impact on the effects of acaricide toxicity on bees [19]. There is also an indirect impact through hive products, recognized as linked to problems in the reproductive biology of queen bees and drones [19], which stands in contrast to the assumption that pesticides used within the hive must be minimally harmful to the bees, while maintaining toxicity to their target organism [20], and shows the role of toxic residues deposited in bee products. Popular acaricides used across the world have been proven to leave residues in hive products. These residues comprise pharmacologically active substances, excipients, or degradation products, and their metabolites. This means that some of these substances could be harmful to the superorganism that comprises the bee colony, hive products, and hive body, or may act synergistically with natural components of bee products present in the hive.

In terms of bee products in the EU, MRL for amitraz and coumaphos in honey are given [21] as 200 μg/kg and 100 μg/kg, respectively. MRL for cymiazole was established at 1000 μg/kg in honey but is no longer included on the list [21] and is not used in bees. For propolis, included among other apiculture products, MRLs have been established for 22 pesticides [11]. It is unlikely that propolis will be taken into consideration as much as honey in terms of establishing official MRLs for active substances present in VMPs, despite the fact that EU law indicates a clear distinction between edible and inedible propolis [22]. It was recently noted by the European Food Safety Authority (EFSA) that the consumption of other bee products, including propolis, is negligible. Therefore, there is no need to generate experimental residue data for these commodities [23]. Such an opinion, however it may relate to food safety issues, raises an alarming problem with regard to bee health.

The aim of this review was to summarize current data on propolis and its products as potential sources of residues of acaricides authorized in the treatment of bee colonies in the EU. Veterinary public health was here a point of reference for legal regulations, bee health, and safety of the hive products in question.

## 2. VMPs Residues in Propolis

Resin used for the production of propolis has been proven to prevent the spread of diseases and parasites in honey bees [24]. Propolis has been described as active enough to show its action against *Varroa destructor*, and was reported to show narcotic and lethal effects [25] towards the parasite. Propolis extracted with ethanol showed a deadly effect on *Varroa* mites [25], and the same was confirmed for topical treatment by spraying with propolis as a natural alternative acaricide [26]. Most recently, it has been confirmed that propolis applied to brood cells before oviposition can influence mites’ parasitization of the bee pupae, decreasing the mites’ survival, and reducing their reproduction [27]. This is probably due to interference with neuronal transmission in mites, prompting their reduced heat production and oxygen consumption [28]. However, some studies have indicated that the varroacidal effect of propolis comes from acaricide residues deposited in propolis as a result of previous treatments [29].

Two factors are implicated in the occurrence of hazards related to acaricidal residues in bee products. Firstly, substances that facilitate dissolution of a drug or chemical greatly enhance the likelihood of absorption [18] and residue occurrence in propolis. Secondly, it has been estimated that, in propolis, pesticides resulting from beekeeping procedures are usually present at higher concentrations than those of environmental origin [30].

Synthetic acaricides used as VMPs in the control of *V. destructor* in honey bees can be divided into three main groups. These include pyrethroids, i.e., flumethrin, fluwalinate and acrinathrin; organophposphorus compounds, i.e., coumaphos, bromfenvinphos; and formamidines, i.e., amitraz and cymiazole [31]. According to their chemical properties, varroacides are divided into fat-soluble (pyrethroids, organophosphorus compounds, amitraz) and water-soluble cymiazole. Most of the synthetic drugs authorized for use in bee colonies consist of a single active substance. However, a complex solution of lipophilic tau-fluvalinate and amitraz for bee-hive strips is available. It was reported that the combination of two lipophilic compounds, even if applied separately, showed an increase of toxicity in both of them [20]. Thus, rotation in the use of VMPs must be an additional factor taken into consideration when toxicity is discussed, with reference to possible synergism in bees and hive products.

When compared to honey, propolis is more prone to residues of fat-soluble active substances. This is due to the higher lipid content in propolis, which varies from 25–50%. Results have confirmed the presence of residues of fat-soluble acaricides in propolis harvested from bee colonies after fumigation or direct contact, or even in propolis-based sweets [30,32]. Fat-soluble ingredients, when introduced to the hive in the form of acaricidal VMPs, are distributed throughout the colony by the bees’ legs and bodies [33].They accumulate in the hive environment, which leads to their constant contact with mites and the build-up of resistance [34]. Bee combs can show acaricidal activity from 3 months to 1 year of application, and resistance to one VMP can build up resistance to another [19]. Residues of authorized and no-longer authorized acaricides, e.g., bromopropylate, have been reported in propolis [32,35,36]. If a lipophilic acaricide is no longer used, residues are gradually diluted through newly produced wax that has not been exposed to the specific acaricide. As shown in the case of bromopropylate in Switzerland, this process is very slow, taking more than two decades [36].

In order to avoid resistance to synthetic varroacidals, thymol and organic acids are used. These are not neutral towards honey bees and their negative effects have been reviewed in detail elsewhere [19]. Organic acids are non-volatile and water soluble. Thymol is volatile, water soluble, and moderately lipophilic [18]. Both are natural plant constituents, and in low concentrations they are present in honey. However, thymol, being a lipophilic acaricide, is among those that pose the main contamination risks for propolis [13].

Other ingredients of natural acaricides that are highly lipid soluble include camphor, menthol, and cineole. Essential oils are naturally present in propolis, and analysis of their residues after treatment can be complicated and inconclusive. In honey they can lead to adverse effects on taste, while residues in wax can render it unsuitable for some uses [37] that may also apply to propolis.

Although research concerning pesticide residues in bee products has been the focus of great interest, with a price index of 47.5% [38], there are only a few scientific reports on residues of synthetic acaricides in propolis (Table 2), and very few analyzing the presence of their degradation products [30].

It is also probable that negative research results have not been published, which obscures the overall picture of the problem. Scarcity of data may also result from the fact that raw propolis is a very complex matrix that requires a very detailed and laborious analytical protocol. The less that propolis is processed the more complex matrix it retains [30]. Therefore it is not routinely investigated in national laboratories that monitor residues in food and food supplements. For some of these residues, e.g., amitraz, metabolites like *N*-2,4-dimethylphenyl-*N*-methyl-formamidine (DMPF) and 2,4-dimethylaniline (DMA) must also be included in the protocol [43]. Studies reporting wax in comparison to beebread and brood found contamination with amitraz at the highest concentrations, followed by coumaphos and tau-fluvalinate [44]. Similar observations were made in Poland regarding residues of VMPs that are illegal in the country. Tau-fluvalinate and coumaphos were present in beeswax and wax foundation that was most possibly made of contaminated imported wax [45]. There is a very high probability that propolis, due to its wax content, can be subject to similar contamination. Lack of routine monitoring of acaricide residues in propolis was noted and reported in the end of the 20th century [33]. It is reflected now at EU level in the databases of the Rapid Alert System for Food and Feed (RASFF), where not even a single case of such a hazard has been reported for years. At the same, time the example of propolis from Uruguay shows an alarming tendency with coumaphos present in two food chain links, i.e., crude propolis [39] and propolis-based product [30]. Moreover, the authors of the latter experiment calculated that ingestion of 10 propolis-based candies contaminated with coumaphos would be enough to reach the Acceptable Daily Intake (ADI) for this pesticide in human. The presence of pesticide residues in propolis were also identified as significant hazards in the Hazard Analysis Critical Control Point (HACCP) system established for the production of energy booster capsules [46].

The use of natural acaricides seems to offer a solution to the problem of residues of synthetic varroacidal VMPs in bee products, and their hazardous toxic effects on bee colonies. However, uncontrolled levels of organic acids and thymol in propolis may lead to changes in its organoleptic features such as smell and taste, which was the case in honey [47]. Long-term application of moderately fat-soluble thymol can lead not only to bees’ death but also to the presence of thymol residues in beehive products, such as propolis [42]. In the EU, thymol is classified as hazardous when ingested [48] and this characteristic of thymol was raised by the EFSA that identified absence of an Acceptable Operator Exposure Level (AOEL) as a critical area of concern [49]. However, binding EU law states that there no MRL for thymol is required in foodstuffs of animal origin [21]. At the same time, thymol residues were reported in propolis from Portugal and Croatia [42,50]. The acaricidal effect of propolis obtained from colonies that have previously been treated with thymol was also reported [29]. Moreover, the application of thymol is not neutral to the health and performance of honey bees. Thymol was proved to interact with other substances acting at the GABA receptor and, similarly to organic acids and synthetic acaricides, can also reduce production of vitellogenin [51]. On the other hand, studies of thymol residues in beeswax showed that only a small proportion of thymol is transferred directly to the bee brood via contaminated wax inside the beehive during larval development [52]. This observation stands in contrast to most synthetic acaricides, in which beeswax plays a main role in the developing bees’ exposure to chemical residue [44]. Considering the above, and with no MRLs established for acaricides in propolis, it would be justified to at least initially implement MRL levels similar to those established for honey [13], although wax would serve as a much more suitable standard. As a propolis component and initial beehive matrix for lipid soluble VMPs, it is assumed to be a vector for chemical residues in the beehive [44]. The published results of research activities can significantly highlight the problem of VMPs in propolis. However, the final confirmation of concerns regarding consumer intake of contaminated propolis is left to the discretion of the EFSA. Sufficiently validated analytical methods based on high-performance liquid chromatography with tandem mass spectrometry (HPLC-MS/MS) are available to quantify residues of pesticides in bee products. To investigate the magnitude of the VMP residues in propolis, a sufficient number of supervised residue trials must be completed, including field and semi-field trials. Moreover, consumer risk assessment should be performed. There are scientific reports available reviewing propolis extraction methods [53], approaches for standardization and quality control of propolis for the purposes of industrial applications [54], and methods for detection of residues in bee products [55,56,57]. Their advantages and disadvantages are described, which can be helpful when establishing unified protocols for accredited laboratories.

This review shows considerable gaps in data regarding residues in propolis of VMPs approved for use in bee colonies in the EU, when compared to the number of reports on residues of environmental origin [38,58], biological properties [59], antimicrobial activity [60,61,62], antioxidant activity [61,63], and chemical composition [64]. In addition to well-known ways of using propolis for the production of so-called over-the-counter (OTC) preparations, supplements, drugs, ointments, and cosmetics [65,66], propolis shows great potential for applications in the medical sector related to treatment of diseases, including Parkinson’s disease [67], allergies [68], cancer [69], or COVID-19 [70]. Importantly, there are numerous emerging fields for its application in animal industries including production, food preservation, and packaging [61,71]. This means that underregulated products may enter the food chain, and the potential impact on public health in terms of inevitable residues should be further explored and monitored.

## 3. Conclusions

1. Propolis is significant, undervalued, and an insufficiently tested source of VMPs residues in the hive.
2. There is an urgent need to legally regulate the status of propolis as an edible product of animal origin.
3. MRLs should be established for VMP residues in propolis, and their inclusion in monitoring programs should be supported at the level of EU and national regulators.
4. Widely studied and publicized bioactive and pro-health properties can be considered only for propolis that is free from VMPs residues.

## Figures and Tables

**Table 1:** VMPs for honey bees authorized for use in EU/EEA [16].

Active Substance	Short Trade Name	Pharmaceutical Form	Country with Authorization
Synthetic Acaricides			
Pyrethroids			
Tau-fluvalinate	Apistan	Bee-hive strip	Austria, Estonia, France, Latvia, The Netherlands, Spain, Sweden
	Gabon PF 90	Bee-hive strip	Slovakia
	Mavrirol	Bee-hive strip	Romania
	M-1 AER	Concentrate for nebulizer solution	Slovakia
Fluvalinate	Apistan	Bee-hive strip	Italy
	Gabon PF	Bee-hive strip	Czechia
	MP 10 FUM	Bee-hive solution	Czechia
	M-1 AER	Bee-hive solution	Czechia
Flumethrine	Bayvarol	Bee-hive strip	Croatia, Bulgaria, Estonia, France, Germany, Hungary, Ireland, Latvia, Poland, Romania, Slovakia, Slovenia, Spain, UK (Northern Ireland)
	Gabon Flum	Bee-hive strip	Czechia
	PolyVar Yellow	Bee-hive strip	Austria, Belgium, Bulgaria, Croatia, Cyprus, Czechia, Denmark, Estonia, France, Germany, Greece, Hungary, Ireland, Italy, Luxembourg, The Netherlands, Poland, Portugal, Romania, Slovakia, Slovenia, Spain, Sweden, UK (Northern Ireland)
	Varostop	Bee-hive strip	Bulgaria, Latvia
Organophosphorous Compounds			
Coumaphos	AB VAR C	Tablets	Bulgaria
	Apifosz	Concentrate for cutaneous solution	Hungary
	CheckMite+	Bee-hive strip	Bulgaria, Croatia, Hungary, Romania,
	Perizin	Concentrate for dip emulsion	Bulgaria
Formamidines			
Amitraz	Amicel Varroa	Solution for bee-hive strip	EU countries
	Apistrip	Bee-hive strip	Poland
	Apitraz	Bee-hive strip	Austria, Bulgaria, Croatia, Czechia, France, Germany, Greece, Hungary, Italy, Portugal, Spain, UK (Northern Ireland)
	Apivar	Bee-hive strip	Austria, Belgium, Bulgaria, Croatia, Cyprus, Czechia, Denmark, Finland, France, Germany, Greece, Hungary, Ireland, Italy, Lithuania, The Netherlands, Norway, Poland, Romania, Slovakia, Slovenia, Spain, Sweden, UK (Northern Ireland)
	Apivartin	Bee-hive strip	Slovakia
	Apiwarol	Bee-smoke stick	Poland
	Avartin B-90	Bee-hive strip	Slovakia
	Biowar	Bee-hive strip	Poland
	Tik-Tak	Cutaneous solution	Hungary
	Varatraz	Solution for bee-hive strip	Romania
	Varidol	Bee-hive solution	Czechia, Slovakia
Complex			
Tau-fluvalinate Amitraz	Varachet Forte	Solution for bee-hive strip	Romania
Natural Acaricides			
Organic Acids			
Sipelghape (formic acid)	AMO Varroxal	Nebuliser suspension	Austria
	Apifor	In-hive use (solution) Cutaneous solution	Austria, France, Germany, Hungary, Italy, Slovenia
	Formicpro	Bee-hive strip	Austria, Belgium, Bulgaria, Croatia, Czechia, Denmark, France, Germany, Greece, Hungary, Ireland, Italy, Lithuania, the Netherlands, Norway, Poland, Portugal, Romania, Slovakia, Slovenia, Spain, Sweden, UK (Northern Ireland)
	Formidol	Bee-hive strip	Czechia, Slovakia
	Furmitom	Bee-hive strip	Bulgaria
	Formivar	Nebulisation solution	Austria, Hungary, the Netherlands, Portugal, Slovenia
	MAQS	Bee-hive strip	Bulgaria, France, Germany, Greece, Hungary, Ireland, Italy, Lithuania, Portugal, Romania, Slovenia, Spain, UK (Northern Ireland)
Sipelghape Iodine	Nosestat	Oral solution	Bulgaria
Oxalic acid	API-Bioxal	Bee-hive solution	Hungary, Latvia, Poland, Slovakia
	Ecoxal	Powder for oral solution	Spain
Oxalic acid dihydrate	API-Bioxal	Bee-hive solution	Austria, Bulgaria, Croatia, France, Hungary, Ireland, Italy, Latvia, Norway, Poland, Portugal, Romania, Slovakia, Slovenia, Spain, UK (Northern Ireland)
	API-Bioxal	Powder for bee-hive solution	Austria, Bulgaria, Croatia, France, Ireland, Italy, Norway, Portugal, Romania, Slovenia, Spain, UK (Northern Ireland)
	Dany’s BienenWohl	Powder and solution for bee-hive dispersion	Austria, Belgium, Bulgaria, Croatia, Cyprus, Czechia, Denmark, Estonia, Finland, France, Germany, Greece, Hungary, Iceland, Ireland, Italy, Latvia, Liechtenstein, Lithuania, Luxembourg, Malta, The Netherlands, Norway, Poland, Portugal, Romania, Slovakia, Slovenia, Spain, Sweden, UK (Northern Ireland)
	Oxuvar	Cutaneous spray, solution	Austria, Belgium, Croatia, Czechia, Germany, Hungary, Italy, The Netherlands, Portugal, Slovakia, Slovenia, UK (Northern Ireland)
	Oxybee	Powder and solution for bee-hive dispersion	Austria, Belgium, Bulgaria, Croatia, Cyprus, Czechia, Denmark, Estonia, Finland, France, Germany, Greece, Hungary, Ireland, Iceland, Italy, Latvia, Liechtenstein, Lithuania, Luxembourg, Malta, The Netherlands, Norway, Poland, Portugal, Romania, Slovakia, Slovenia, Spain, Sweden, UK (Northern Ireland)
Oxalic acid Sipelghape	VarroMed	Bee-hive dispersion	Austria, Belgium, Bulgaria, Croatia, Cyprus, Czechia, Denmark, Estonia, Finland, France, Germany, Greece, Hungary, Iceland, Ireland, Italy, Latvia, Liechtenstein, Lithuania, Luxembourg, Malta, The Netherlands, Norway, Poland, Portugal, Romania, Slovakia, Slovenia, Spain, Sweden, UK (Northern Ireland)
Thymol and Thymol-Based			
Thymol	Apiguard	Bee-hive gel	Austria, Belgium, Bulgaria, Croatia, Czechia, Estonia, France, Germany, Greece, Hungary, Ireland, Italy, Latvia, Luxembourg, The Netherlands, Poland, Romania, Slovakia, Spain, Sweden, UK (Northern Ireland)
	Apiguard Multidose	Bee-hive gel	France, Ireland, Italy, Poland, Spain, Sweden,
	Thymovar	Bee-hive strip	Austria, Belgium, Croatia, Cyprus, Czechia, France, Germany, Greece, Hungary, Italy, The Netherlands, Poland, Portugal, Romania, Slovakia, Slovenia, Spain, UK (Northern Ireland)
Thymol Cineole Camphor racemic Levomenthol	Apilife Var	Bee-hive strip	Austria, Belgium, Bulgaria, France, Greece, Italy, Portugal, Romania, Slovenia, UK (Northern Ireland)
Thymol Eucalyptus oil Camphor racemic Levomenthol	Apilife Var	Bee-hive strip	Slovakia
Thymol Eucalyptus oil Camphor Natural menthol	Api Life Var	Inhalation vapour tablet	Romania
Pepper mint Thymol	Ecostop	Bee-hive strip	Bulgaria, Hungary
Taraxaci herba Thyme Achillea millefolium flower Ocimum basilicum	Protofil	Oral solution	Romania
d-camphor (1R,2S,5R)-5-methyl-2-(1-methylethyl)-cyclohexanol Eucalyptus oils Thymols	Api Life Var	Bee-hive strip	Croatia, Hungary, Poland
Other Drugs			
Verilopam ^1^	European Medicines Agency (EMA) Verification	Effervescent granules + film-coated tablet	The Netherlands

^1^ Lack of detailed information.

**Table 2:** Reports of the presence or absence of residues in propolis after the use of synthetic acaricides that are authorized in EU.

**Acaricide (Active Substance)**	**Country of Origin of Propolis or Propolis-Based Product Analyzed**	**Reference**
coumaphos	Croatia	[35]
coumaphos	Uruguay	[39]
coumaphos	Uruguay	[40]
coumaphos	Uruguay	[30]
coumaphos	Argentina	[30]
coumaphos ^1^	Brazil	[30]
fluvalinate	Poland	[41]
fluvalinate	Switzerland	[32]
flumethrin	Switzerland	[32]
flumethrin ^1^	Portugal	[42]

^1^ Not Detected.

## Data Availability

Not applicable.
